# Pyogenic Spondylitis Due to Erysipelothrix rhusiopathiae Infection: A Case Report

**DOI:** 10.7759/cureus.92532

**Published:** 2025-09-17

**Authors:** Yusuke Oshita, Takeshi Eguro, Satoshi Kimura, Keikichi Kawasaki, Yoshifumi Kudo

**Affiliations:** 1 Department of Orthopaedic Surgery, Showa Medical University Northern Yokohama Hospital, Yokohama, JPN; 2 Department of Orthopaedic Surgery, Kikuna Memorial Hospital, Yokohama, JPN; 3 Department of Laboratory Medicine and Central Clinical Laboratory, Showa Medical University Northern Yokohama Hospital, Yokohama, JPN; 4 Department of Orthopaedic Surgery, Showa Medical University School of Medicine, Tokyo, JPN

**Keywords:** case report, erysipelothrix rhusiopathiae, matrix-assisted laser desorption ionization-time of flight (maldi-tof), pyogenic spondylitis, spinal infection, zoonotic bacterial infections

## Abstract

The incidence of pyogenic spondylitis is growing worldwide, but that caused by zoonotic bacteria is not well-reported. *Erysipelothrix rhusiopathiae* infections of the spine are rare. We report a case of spondylitis caused by *E. rhusiopathiae* in a patient without a history of livestock industry work. An 82-year-old retired Japanese man experienced vomiting, loss of consciousness, and incontinence during dinner. Subsequently, an ambulance was dispatched, and he was transferred to a local general hospital. Upon admission, brain computed tomography and magnetic resonance imaging revealed no abnormalities, and echocardiography ruled out infective endocarditis. The patient presented with a fever of unknown origin and received piperacillin/tazobactam. Seven days later, a blood culture test identified *E. rhusiopathiae*, leading to a diagnosis of bacteremia caused by this organism. The treatment was then switched to ampicillin. Despite this, the low back pain appeared and worsened. Lumbar MRI detected spondylitis, necessitating spinal surgery. Following the procedure, inflammation and pain were markedly reduced, and no recurrence was noted after one year and four months. Notably, the patient had no history of animal contact but resided in a home heavily littered with garbage, reportedly with rats present. Therefore, the unsanitary conditions may have been a contributing factor to a rare bacterial infection of the spine.

## Introduction

The number of older and nutritionally compromised patients has been increasing, increasing the number of cases of bacterial infections such as pyogenic spondylitis [1-3]. The increasing incidence has been attributed to multiple factors, including chronic diseases and invasive spinal procedures [1], the aging population [2], and immunocompromising conditions [3]. In Japan, the annual incidence rate of pyogenic spondylodiscitis has been reported to range between 2 and 7 per 100,000 population [4].

The predominant causative organisms of pyogenic spondylitis are Gram-positive cocci, particularly *Staphylococcus aureus* [1,3], followed by *Streptococcus*, *Enterococcus species* [3], and Gram-negative bacilli such as *Escherichia coli*, *Klebsiella*, and *Pseudomonas aeruginosa* [3]. In contrast, infections caused by Erysipelothrix rhusiopathiae, a zoonotic, rod-shaped, Gram-positive bacterium, are rarely reported. Only a few cases have been published worldwide [5-9].

*E. rhusiopathiae* is of particular interest because it is a zoonotic bacterial infection, transmitted from animals to humans, yet spinal involvement has rarely been reported. In general, zoonotic bacterial infections have been reported in livestock workers and farmers. In this study, we report a case of spondylitis caused by* E. rhusiopathiae*, in which the patient had no history of employment in such occupations and no experience keeping pets, indicating an absence of direct animal contact. However, based on the work history of a woodcraft artisan, this patient resided in a home littered with garbage, with no apparent cleanup efforts. Although the route of infection was unclear, we encountered a case in which E. rhusiopathiae bacteremia led to the development of spondylitis. Thus, we assume that unsanitary living conditions might be a risk factor for such infections.

We herein report a rare case of pyogenic spondylitis caused by *E. rhusiopathiae*, underscoring the critical role of matrix-assisted laser desorption/ionization time-of-flight mass spectrometry (MALDI-TOF MS) in the rapid diagnosis of uncommon bacterial pathogens.

## Case presentation

**Figure 1:**
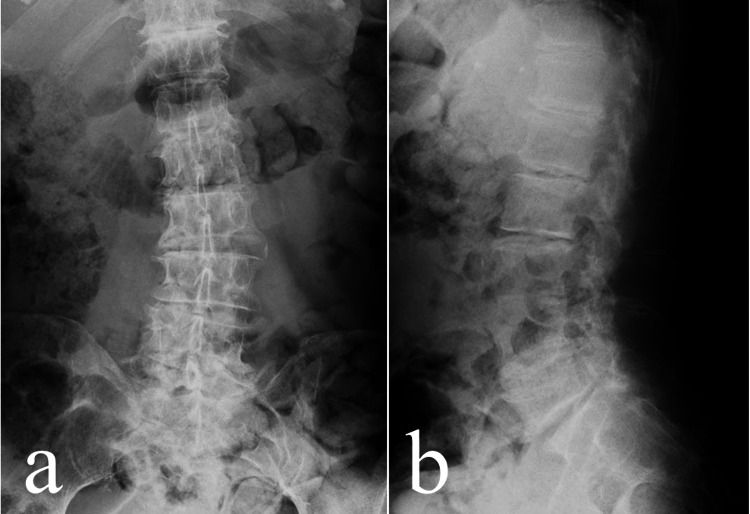
Radiographic image at the first hospital visit a. Anterior-to-posterior view; b. Lateral view Radiographs demonstrated only age-related changes without any abnormal findings

An 82-year-old retired Japanese man (height, 167 cm; weight, 53.2 kg) experienced sudden vomiting, loss of consciousness, and incontinence during dinner. The patient was then transported by ambulance to a local general hospital. Six days earlier, he had experienced pain in the left sciatic nerve. Radiographic examination at the initial visit showed age-related degenerative changes (Figure 1).

The patient had comorbidities, including diabetes mellitus, hypertension, asbestos lung, and bipolar head arthroplasty of the right hip joint following a femoral neck fracture. No dental cavities or full dentures were found. The body temperature was 37.8 °C. Brain computed tomography (CT) and magnetic resonance imaging (MRI) showed negative findings. Laboratory data revealed a white blood cell (WBC) count of 7,800/μL (reference range: 3,300-8,600/μL; neutrophils, 89.3% (42.4-75.0%); lymphocytes, 3.8% (16.5-49.5%); eosinophils, 0.4% (0.0-8.5%); basophils, 0.1% (0.0-2.5%); monocytes, 6.4% (2.0-10.0%)), C-reactive protein (CRP) level of 10.85 mg/dL (reference range: <0.14 mg/dL), and glycated hemoglobin (HbA1c) of 7.9% (reference range: 4.6-6.2%). Urinary tract infection and COVID-19 infection were ruled out. Echocardiography revealed no evidence of infective endocarditis. Whole-body CT did not reveal the source of the fever.

The patient was diagnosed with a fever of unknown origin and was treated with piperacillin/tazobactam (PIPC/TAZ). The next day, the fever spiked to 40.0 °C, and the vital signs were: P 93/min, BP 134/43 mmHg, RR 16/min, SpO₂ 97%. After three days’ admission, the body temperature decreased to 37.8 °C, with a CRP level of 23.53 mg/dL and a WBC count of 10,400/μL (neutrophils, 89.3%; lymphocytes, 5.0%; eosinophils, 0.1%; basophils, 0.2%; monocytes, 6.4%). Gram-positive rods were detected after seven days of blood culture.

**Figure 2:**
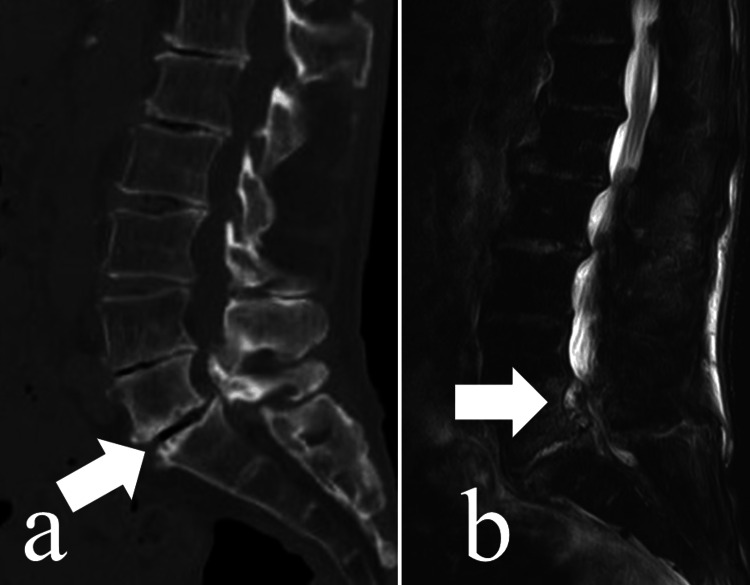
Preoperative CT and MRI images a. CT before surgery. Lytic lesions were detected in the L4/5 intervertebral disc (white arrow). b. MRI before surgery. An abscess was detected at L4/5 (white arrow).

Using a mass spectrometry technique, especially the MALDI-TOF mass spectrometry system, by Bruker Daltonics (Yokohama, Japan) with the Biotyper software, version 3.1 (Bruker Daltonics), the isolate was identified as *E. rhusiopathiae*. Antimicrobial susceptibility testing showed that the isolate was susceptible to PCG (penicillin G), ABPC (ampicillin), MINO (minocycline), CEZ (cefazolin), CTM (cefotiam), SBT/CPZ (sulbactam/cefoperazone), FMOX (flomoxef), EM (erythromycin), CLDM (clindamycin), FOM (Fosfomycin), CPFX (ciprofloxacin), LVFX (levofloxacin), CFPM (cefepime), and MEPM (meropenem), but resistant to GM (gentamicin) and VCM (vancomycin). According to the results of susceptibility testing, the antibiotic was changed to ABPC. Lower back pain was observed during treatment. Although radiography showed almost normal findings, CT of the lumbar spine was performed and revealed a lytic lesion at the L4/5-disc level. For further evaluation of this lesion, enhanced MRI was subsequently performed, which demonstrated discitis and epidural abscesses (Figure 2).

After being diagnosed with spondylitis, the patient was transferred to a university hospital. The day of the transfer, a board-certified dermatologist conducted a thorough skin examination and confirmed the absence of any skin infection, such as scabies, ticks, or lice. The day after the transfer, L4/5/S1 laminoplasty surgery was performed. During the surgery, a subdural abscess compressing the dura mater of the nerve root was removed. Postoperatively, the pain decreased. Seven days after surgery, the inflammatory parameters improved: CRP levels decreased to 0.79 mg/dL and WBC count to 3,470/μL (neutrophils, 69.1%; lymphocytes, 20.5%; eosinophils, 3.7%; basophils, 1.2%; monocytes, 5.5%), with no symptoms of infection. On hospital day 24, after resolution of inflammation, the intravenous ampicillin/sulbactam (ABPC/SBT) was switched to oral clindamycin (CLDM) 450 mg/day. The patient did not experience spinal complications such as palsy or dysuria. Twenty days after the surgery, the patient was transferred to a rehabilitation hospital. Ninety-two days after surgery, the patient was discharged and could walk unaided. During hospitalization, the family hired a cleanup company to maintain cleanliness at the patient’s home.

On postoperative day 125, MRI revealed no abscesses. CT imaging showed a trend toward union. At 195 days post-surgery, radiography indicated slight disc height narrowing, yet the patient remained asymptomatic. Five months post-surgery, no typical changes were observed. One year and six months post-surgery, there were no symptoms of pyogenic spondylitis recurrence.

The overall clinical course of this case, including clinical events, antibiotic administration, diagnostic procedures, and outcomes, is summarized in Table 1. This case underscores the clinical significance of recognizing the progression from fever of unknown origin to bacteremia, identification of *E. rhusiopathiae*, and subsequent spinal infection, which may facilitate earlier diagnosis and management in similar cases.

**Table 1:** Clinical timeline of this case Timeline of this case, showing clinical events and antimicrobial agents used. Clinical course of this case, showing progression from fever of unknown origin to bacteremia and spinal infection. PIPC/TAZ, piperacillin/tazobactam; ABPC, ampicillin; ABPC/SBT, ampicillin/sulbactam; CLDM, clindamycin.

Time Period	Event Description	Antimicrobials
Six days before	Sciatic nerve pain developed	
Day 0	Loss of consciousness: Fever of unknown origin	PIPC/TAZ
Day 7	Enhanced lumbar MRI: Diagnosed pyogenic spondylitis	ABPC
Day 12	Transferred from a general hospital to a university hospital	ABPC/SBT
Day 13	Spinal surgery	
Day 24	Resolution of inflammation	Oral CLDM
Day 34	Transferred from the university hospital to a rehabilitation hospital	
Day 106	Discharge to home	
Day 117	Outpatient follow-up: No symptoms	Terminate antimicrobials
Day 138	Lumbar MRI: No evidence of recurrence	
Day 481	Final check-up at this point: No symptoms	

## Discussion

Recent studies from South Korea and Germany have shown an increasing incidence of pyogenic spondylitis [1-3]. Reported rates range from 5.4 per 100,000 in Germany in 2005 to 11.0 per 100,000 in 2019, representing a two-fold increase [2], and from 22.9 to 35.8 per 100,000 in South Korea between 2010 and 2019, representing a 1.5-fold increase [1].

Rostamian et al. reviewed 62 case reports of *E. rhusiopathiae* infection [5]. The review describes only four cases from Japan, of which three were infective endocarditis. Of these three patients, two were fishermen [10,11] and one was a voluntary worker in a school for handicapped children [12] (Table 2). The remaining case was a soft tissue infection of the right hand caused by a cat bite [13].

**Table 2:** Erysipelothrix rhusiopathiae-induced Infectious endocarditis reports from Japan

Age	Sex	Occupation	
58	Male	Fisherman	2008 Yamamoto et al. [10]
67	Male	Fisherman	2011 Haradaet al.[11]
42	Female	Voluntary worker	2013 Miura et al. [12]

Romney et al. reported that* E. rhusiopathiae* is sensitive to penicillin and carbapenem but resistant to vancomycin [14]. In this case, although the isolate was susceptible to ABPC, the regimen was switched to intravenous ampicillin/sulbactam (ABPC/SBT) after transferring due to the hospital formulary policy, and oral CLDM was added based on the results of antimicrobial susceptibility testing.

The patient had no history of animal contact other than exposure to rats and had not been bitten. Hypertension and diabetes were the underlying comorbidities. According to the patient, he had not changed his bed linen in these years; he deduced that brown rats might have settled in his room, and he saw cockroaches daily. Moreover, the patient bathed once a week with reused water. Narvaez et al. also report a homeless spondylosis case without any exposure to any animal contact [6]. Thus, we assume that sanitation is important to prevent this infection. A review reported that 21 out of 62 cases (34%) presented with skin lesions and 23 cases (37.1%) with heart valve infections [5]. However, the present case exhibited neither skin lesions nor infective endocarditis.

In general, some reports state that pyogenic spondylitis can arise from dental cavities [15,16], but no dental cavities were identified in this case. In contrast to the typical cause of pyogenic spondylitis, the fact that *E. rhusiopathiae* is not part of the oral flora might have an impact. Interestingly, the other reports of *E. rhusiopathiae* infection cases do not discuss the oral infections [5,7-13]. A few case reports have described spinal infections caused by* E. rhusiopathiae* (Table 3).

**Table 3:** Reports of Erysipelothrix rhusiopathiae-induced infectious spondylitis COPD, chronic obstructive pulmonary disease.

Age	Sex	Occupation	Risk	Infection Site	Treatment	
67	Female	Not specified	Alcoholic/injection drug use	L3 osteomyelitis	Antibiotics	2001 Romneyet al. [5]
62	Male	Farmar	Diabetes mellitus	T5/6 epidural abscess	Surgical drainage	2009 Andrychowski et al. [8]
62	Male	Farmar	Diabetes mellitus	L2/3 epidural abscess	Surgical drainage	2014 Upapan et al*.* [7]
48	Male	Not specified	Puncture injury to his left index finger from the barb of a sea fish	L5/S osteomyelitis	Antibiotics	2018 Lorenz et al. [9]
71	Male	Homeless	Diabetes mellitus, chronic obstructive pulmonary disorder (COPD), chronic leukocytosis, and dirty daily life	Th6/7	Drainage	2022 Narvaezet al. [6]
82	Male	Retired	Dirty daily life/Diabetes mellitus	L5/S Pyogenic spondylitis subdural abscess	Surgical drainage	2025 Our case

Notably, Upapan et al. reported a case of a psoas abscess with *E. rhusiopathiae* without endocarditis [7], which was treated with surgical drainage. Moreover, Andrychowski et al. reported thoracic spondylitis caused by *E. rhusiopathiae* [8], in which the abscess was drained. In our case, the epidural abscess caused sciatic nerve pain, drainage, and nerve root decompression. Importantly, symptoms of lower back and sciatic nerve pain disappeared immediately after spinal surgery. In this case, we assumed that surgical drainage with nerve root decompression. Systematic reviews of pyogenic spondylitis have also reported that prompt surgical intervention is crucial for the avoidance of lumbar complaints after surgery [17].

Until recently, *E. rhusiopathiae* infections have not been frequently reported. As we report in this paper, MALDI-TOF testing will enable us to identify these zoological microorganisms more easily.

## Conclusions

We successfully treated a patient with spondylitis secondary to bacteremia caused by *E. rhusiopathiae*. As far as we could ascertain, this is the first reported case of* E. rhusiopathiae*-induced spondylitis from Japan. Although limited to a single case, this report highlights that* E. rhusiopathiae* infection can pose a potential risk in compromised hosts and provides educational value for clinicians in recognizing and managing such severe infections.
